# Performance of euroscore ii compared with its predecessors in octogenarian patients undergoing aortic valve replacement

**DOI:** 10.1186/1749-8090-10-S1-A352

**Published:** 2015-12-16

**Authors:** Daniel HernandezVaquero, Rocio Diaz, Ruben AlvarezCabo, Carlos Morales, Jacobo Silva

**Affiliations:** 1 Cardiac Surgery Department, Central University Hospital of Asturias, Oviedo, Spain

## Background/Introduction

Logistic EuroSCORE overestimates the risk profile of octogenarians undergoing aortic valve replacement by traditional surgery. EuroSCORE II, that was created in an attempt to improve this previous version, has been evaluated in the general population. However, to our knowledge, there are no studies evaluating the predictive performance of EuroSCORE II in the elderly population undergoing surgery for aortic valve replacement despite the fact that the majority of patients receiving transcatheter techniques are octogenarians and this new version is being used for the selection of high-risk surgical patients.

## Aims/Objectives

Our objective was to perform a complete evaluation of EuroSCORE II in octogenarian patients undergoing aortic valve replacement and compare it with its predecessors.

## Method

All octogenarians who underwent aortic valve replacement between 2009 and 2015 were analyzed. EuroSCORE II was assessed calculating discrimination using the area under the ROC curve (AROC), and calibration using the Hosmer-Lemeshow (HL) test and the risk adjusted mortality ratio (RAMR) of the model. The same analysis was performed by risk quartiles.

## Results

482 octogenarian patients underwent aortic valve replacement in our center during the study period. Discrimination of EuroSCORE II was excellent, AROC=0,91, and better than its previous versions, AROC for logistic EuroSCORE =0,81 and for additive EuroSCORE=0,80. Calibration was poor due to underestimation of the mortality risk. (p for Hosmer-Lemeshow test of 0,03 and risk adjusted mortality ratio=8,9/6,1=1,46). However, analyzing by quartiles, EuroSCORE II has almost perfect calibration and discrimination for all patients except for those with the highest risk. In the fourth quartile of risk, EuroSCORE II markedly underestimates the mortality risk. In these high-risk patients, the best calibration is reached using the logistic EuroSCORE (p for HL test 0,23 and RAMR =29,2/24,91= 1,17).

## Discussion/Conclusion

High risk patients can be detected using EuroSCORE II due to its excellent discrimination power. However, once a patient is selected as high-risk (EuroSCORE II > 6,5), logistic EuroSCORE should be used to calculate the risk of mortality.

